# Prevalence of Third-Party Data Tracking by US Hospital Websites

**DOI:** 10.1001/jamanetworkopen.2021.26121

**Published:** 2021-09-22

**Authors:** Joshua D. Niforatos, Alexander R. Zheutlin, Jeremy B. Sussman

**Affiliations:** 1 Department of Emergency Medicine, The Johns Hopkins School of Medicine, Baltimore, Maryland; 2 Department of Internal Medicine, University of Utah School of Medicine, Salt Lake City; 3 Department of Internal Medicine, Division of General Medicine, University of Michigan, Ann Arbor; 4 Center for Clinical Management Research, Veterans Affairs Ann Arbor Healthcare System, Ann Arbor, Michigan; 5 Institute for Healthcare Policy and Innovation, University of Michigan, Ann Arbor

## Abstract

This cross-sectional study compares the prevalence of third-party data tracking on websites of the largest and top-rated nonprofit and for-profit hospitals in the US.

## Introduction

Web tracking occurs when third parties collect, store, and share information related to a website visitor’s activity.^1^ Hospital websites may reveal sensitive information about an individual’s health conditions and concerns often without their knowledge. We characterized data tracking on US hospital websites to understand potential patient privacy risks.

## Methods

We conducted a cross-sectional study of publicly available website data tracking on June 23, 2021. This study did not require institutional review board approval based on the criteria of 45 CFR §46 because it was not regulated as human participant research. Three categories of US hospitals were included in this study (Table): the 20 largest for-profit hospitals and the 21 largest nonprofit and governmental hospitals (nonprofit) according to the 2019 American Hospital Association annual survey and the 20 best hospitals according to the *US News & World Report* (*USNWR*) 2020-2021 Best Hospitals Honor Roll. More details are given in the eMethods in the Supplement. This study followed the Strengthening the Reporting of Observational Studies in Epidemiology (STROBE) reporting guideline.

**Table.** Third-Party Data Tracking on Websites of Major US Hospitals

Type of tracking	*USNWR* top hospitals (n = 20)^a^	Largest nonprofit hospitals (n = 20)	Largest for-profit hospitals (n = 20)	Total
Advertisement trackers				
No. (%)^b^	20 (100)	21 (100)	20 (100)	61 (100)
Median (IQR)	9.0 (4.8-11.3)	6.0 (3.0-9.0)	5.0 (3.0-6.3)	6.0 (4.0-9.0)^c^
Third-party cookies				
No. (%)^b^	20 (100)	18 (86)	17 (85)	55 (90)
Median (IQR)	11.5 (4.0-22.0)	4.0 (1.0-10.0)	3 (2.0-5.8)	4 (2.0-16.0)^c^
Session recording services	7 (35)	5 (24)	2 (10)	14 (23)
Information provided to Facebook, No. (%)^b^	14 (70)	14 (67)	12 (60)	40 (66)
Information provided to Google analytics, No. (%)^b^	17 (85)	18 (86)	19 (95)	54 (89)

Abbreviations: IQR, interquartile range; *USNWR*, *US News & World Report*.

^a^ Hospitals on the *USNWR* 2020-2021 Best Hospitals Honor Roll.

^b^ Percentages are based on row totals.

^c^ Median and IQR based on summative data.

For each hospital, we gathered information regarding their website’s data tracking using Blacklight,^2^ which monitors website surveillance scripts (eMethods in the Supplement).^3^ We collected information on advertisement tracking, third-party cookies, session recording services, and Facebook and Google tracking software. Data were analyzed descriptively.

## Results

A total of 61 hospital websites were included. All hospitals used advertisement trackers, and most (55 [90%]) used third-party cookies (Table). Session recording services were found on 14 hospital websites (23%), whereas 40 websites (66%) used Facebook tracking software and 54 (89%) used Google tracking software (Table).

The median values for both advertisement trackers and third-party cookies were highest among the *USNWR* best hospitals (trackers: 9.0 [interquartile range (IQR), 4.8-11.3]; cookies: 11.5 [IQR, 4.0-22.0]), followed by nonprofit hospitals (trackers: 6.0 [IQR, 3.0-9.0]; cookies: 4.0 [IQR, 1.0-10.0]) and for-profit hospitals (trackers: 5.0 [IQR, 3.0-6.3]; cookies: 3.0 [IQR, 2.0-5.8]) (Table). The proportions of websites with session recording services and Facebook tracking software were highest among the *USNWR* best hospitals (7 [35%] and 14 [70%], respectively), whereas the proportion of websites with Google tracking software was greatest among for-profit hospitals (19 [95%]).

## Discussion

All hospital websites in the current analysis used advertisement trackers, and most used third-party cookies and provided user information to Facebook and Google. The websites for the *USNWR* best hospitals used more advertisement trackers and third-party cookies than the websites for the other hospital categories. A higher proportion of *USNWR* best hospitals also used session recording services and provided user information to Facebook.

Patients often use hospital websites when seeking health care services. In this process, patients create a digital profile that gives information on the patient’s location, demographic characteristics, and disease status, giving hospitals and other organizations who receive their data the ability to construct health profiles of individuals that can be used for targeted advertising and monitoring or to better understand information-seeking habits of the website users.

The *USNWR* best hospital websites had substantially more tracking than the nonprofit and for-profit hospital websites. Hospital rankings may inform and influence consumer behavior and are often used to justify care locations by patients.^4,5,6^ Furthermore, patients with rare diseases or diseases refractory to common therapies in particular may benefit from the subspecialty care at top hospitals but also may be at increased risk of identification.

The current study is limited by the use of only 1 method to examine website tracking; other algorithms for monitoring data privacy may vary. In addition, we cannot comment on how the data are used or how aware hospital leadership is of the tracking. Given the substantial amount of information patients may be providing to hospital systems through their websites, future research is warranted regarding how this information is used, whether protected health information is inadvertently created, and whether patients are provided easily accessible and understandable up-front consumer privacy disclosures.

## Notes

Supplement.
eMethods
