# Supplementary material for: Prevalence of Third-Party Data Tracking by US Hospital Websites
Source: JAMA Netw Open. 2021 Sep 22;4(9):e2126121. doi: 10.1001/jamanetworkopen.2021.26121 (PMC8459186; doi:10.1001/jamanetworkopen.2021.26121)
Supplement: Supplement. — eMethods [file jamanetwopen-e2126121-s001.pdf]

Supplemental Online Content

Niforatos JD, Zheutlin AR, Sussman JB. Prevalence of third-party data tracking by US Hospital websites. *JAMA Netw Open.* 2021;4(9):e2126121.
doi:10.1001/jamanetworkopen.2021.26121

eMethods

This supplemental material has been provided by the authors to give readers additional information about their work.

© 2021 Niforatos JD et al. *JAMA Network Open.*

eMethods

We conducted the cross-sectional study of publicly available website data tracking was conducted on June 23, 2021. Three categories of U.S. hospitals were used in this study: top-ranked hospitals, for-profit hospitals, and not-for-profit (NFP) hospitals. Top-ranked hospitals were identified using the U.S. News and World Report (USNWR) 2020-21 Best Hospitals Honor Roll, while the largest for-profit and NFP hospitals were identified using the latest available year of the American Hospital Association (AHA) Annual Survey (2019). More specifically, hospitals in the 2019 AHA survey were identified using the following variables: "AHA Identification Number (ID)," "Hospital name (MNAME)," "Control Code - type of authority responsible for establishing policy concerning overall operation of the hospitals (CNTRL)," and "Total hospital beds (HOSPBD)". The CNTRL variable was further broken down into NFP vs for-profit status. For-profit includes all categories under the umbrella term "Investor-owned, for-profit", while NFP was combined into a single category which included all categories under the following umbrella terms "Government, nonfederal," and "Government, federal," "Non-government, not-for profit (NFP)." Hospitals that had both substantial for-profit and NFP ownership were categorized as for-profit, which made Tulane Health System a for-profit organization in this study. The top 20 largest hospitals in each latter category were included for analysis. The number 20 was chosen given the USNWR reports the top 20 hospitals. A list of included hospitals can be found at the GitHub repository: https://github.com/reverendofdoubt/thirdpartydatatracking_jamaNO_2021.

For the NFP hospitals, any hospital that was already in the USNWR was not counted towards the data for NFP category. For completeness sake, these hospitals were still listed in the NFP

© 2021 Niforatos JD et al. *JAMA Network Open.*

category according to size though with asterisks to indicate these data is not included in this category. Similarly, for the for-profit category, a number of hospitals listed in this category are part of one for-profit system and share the same landing internet home page. Each hospital system was thus only counted once, and are indicated with asterisks.

Thus, some categories in the raw data may add up to more than 20 hospitals, but only the first 20 hospitals that were unique were counted in each category.

We inputted the main page of each hospital website URL included in the study through Blacklight to identify website data tracking. Blacklight is an internet program created by the not-for-profit organization The Markup,[6] which monitors website surveillance scripts. Blacklight works by visiting each website with a headless browser running custom software built by The Markup. The main page of the website is analyzed, as well as randomly selected pages from the website. Each page is scanned for known types of privacy violations and thus data collected is for ad tracking, third-party cookies, session recording services, Facebook tracking, and Google Analytics tracking. More information on the methodology and creation of Blacklight, is available at the developer's website: https://themarkup.org/blacklight/2020/09/22/how-we-built-a-real-time-privacy-inspector. Previous research using Blacklight include Zheutlin et al 2021 (DOI: 10.1007/s11606-021-06695-8). Similar software, webXray, has been used in similar research published in JAMA regarding data tracking of covid-19 related websites (DOI:10.1001/jama.2020.16178). Blacklight reports the average popular website has 7 ad-trackers and 3 third-party cookies.

© 2021 Niforatos JD et al. *JAMA Network Open.*

Data was described using descriptive statistics. The Strengthening the Reporting of Observational Studies in Epidemiology (STROBE) reporting guideline for cohort studies were followed in this report.

© 2021 Niforatos JD et al. *JAMA Network Open.*
